# Progression-directed ablative radiotherapy improves event-free survival in oligoprogressive NSCLC

**DOI:** 10.3389/fonc.2026.1766544

**Published:** 2026-05-25

**Authors:** Lorenzo De Sanctis, Riccardo Ray Colciago, Giulia Rossano, Matteo Ferrari, Matteo Mombelli, Ilenia Manno, Federica Ferrario, Sara Saufi, Valeria Faccenda, Denis Panizza, Gaia Passarella, Stefania Canova, Diego Luigi Cortinovis, Stefano Arcangeli

**Affiliations:** 1 Medicine and Surgery Department, University of Milan Bicocca, Milano, Italy; 2 Radiation Oncology, Fondazione Istituto di Ricovero e Cura a Carattere Scientifico (IRCCS) San Gerardo Dei Tintori, Monza, Italy; 3 Medical Physics, Fondazione Istituto di Ricovero e Cura a Carattere Scientifico (IRCCS) San Gerardo Dei Tintori, Monza, Italy; 4 Medical Oncology Unit, Fondazione Istituto di Ricovero e Cura a Carattere Scientifico (IRCCS) San Gerardo dei Tintori, Monza, Italy

**Keywords:** event-free survival, local control, oligoprogressive non-small cell lung cancer, progression-directed radiation therapy, systemic therapy

## Abstract

**Introduction:**

To assess the efficacy of progression-directed radiation therapy (PDRT) in patients with oligoprogressive non-small cell lung cancer (NSCLC), with particular focus on disease progression and changes in systemic therapy.

**Materials and methods:**

From January 2020 to October 2025, a retrospective single-center analysis was conducted at our Institution, including NSCLC patients treated with PDRT for oligoprogressive disease. Oligoprogression was defined as progression involving fewer than five extracranial lesions or a total intracranial disease volume of ≤14 cc, following an initial response to systemic treatment. The primary endpoint was event-free survival (EFS), defined as the occurrence of any of the following: change in systemic therapy, progression within 6 months, or development of >3 progressive lesions. Multivariate Cox regression models were applied to identify predictors of oncological outcomes.

**Results:**

Eighty-seven patients were included, with a median age of 68 years (range: 43–87) and a median follow-up of 14 months. Adenocarcinoma was the predominant histology (75 cases, 86.2%), followed by squamous cell carcinoma (12 cases, 13.8%). PDRT was administered after first-line systemic therapy in 61 patients (70.1%). At the last assessment, 12 (13.8%) patients achieved a complete response, 29 (33.3%) a partial response, and 46 (52.9%) stable disease. The median time to event, death or last follow-up was 5 months (range: 1–48), with a one-year actuarial EFS rate of 52.1% (95% CI: 46.4–57.8%). Median time to next treatment (TTNT) was 8 months (range: 1–68), while median progression-free survival was 5 months (range: 1–39). On multivariate analysis, both PDRT directed at the primary tumor (HR = 0.28, 95% CI: 0.12–0.66; p < 0.01) and achieving a complete response prior to oligoprogression (HR = 0.31, 95% CI: 0.10–0.95; p = 0.04) were significantly associated with improved EFS. Conversely, chemotherapy use (HR = 2.33, 95% CI: 1.21–4.48; p = 0.02) and larger CTV volumes (HR = 1.01, 95% CI: 1.0004–1.0111; p = 0.03) were associated with worse outcomes.

**Conclusions:**

PDRT achieved a median EFS of 5 months and extended the median time to next systemic therapy to 8 months. These findings suggest that PDRT may represent an effective approach to maintaining disease control and postponing systemic treatment in oligoprogressive NSCLC. Larger, prospective studies with extended follow-up are warranted to confirm these results.

## 1 Introduction

Since Hellman and Weichselbaum first introduced the concept of oligometastatic disease (1), the traditional assumption that the presence of metastases uniformly portends a poor prognosis has been reconsidered. Their seminal hypothesis framed metastatic dissemination as a biological spectrum, suggesting that some patients may harbor a limited metastatic potential amenable to curative-intent local treatments. In parallel with advances in systemic therapies, several local ablative approaches—including surgery, radiotherapy (RT), and radiofrequency ablation—have demonstrated encouraging oncological outcomes (2–4). This paradigm shift has been reinforced by evidence from multiple prospective randomized trials that have demonstrated improvements in both progression-free survival (PFS) and overall survival (OS) across various tumor types (5–10).

Among these local modalities, modern RT offers several advantages as a non-invasive technique capable of delivering high doses of radiation over a limited number of sessions, enabling the simultaneous treatment of multiple metastatic sites with minimal disruption to ongoing systemic therapy. Stereotactic body radiotherapy (SBRT), in particular, provides ablative doses with high conformity and steep dose gradients, allowing for the safe targeting of lesions in diverse anatomic locations. While RT has proven effective in managing oligometastatic non-small cell lung cancer (NSCLC), its role in cases of progressive or extensive metastatic disease remains uncertain (11). In the absence of reliable biomarkers to characterize disease progression patterns, therapeutic decisions are primarily guided by imaging and biopsy findings. However, as systemic therapies become increasingly effective, a growing number of patients exhibit mixed patterns of response due to tumor heterogeneity, in which most lesions remain controlled while a limited number progress due to acquired resistance. This clinical scenario has generated increasing interest in the potential of RT to eradicate resistant clones and to prolong the benefit of ongoing systemic treatment.

The term oligoprogression—introduced in the literature in 2012 (12, 13)—refers to a clinical condition in which a limited number of metastatic lesions progress despite otherwise controlled systemic response. Although there is no universally accepted definition, most studies use a threshold of three to five progressing metastatic sites (4, 14). In this context, the intent of RT is to maintain disease control and to delay the initiation of a new systemic treatment until more widespread progression occurs. Emerging retrospective evidence, particularly from the era of immune checkpoint inhibitors and tyrosine kinase inhibitors used in patients with tumors harboring actionable genomic alterations, suggests that progression-directed radiotherapy (PDRT) may effectively suppress resistant lesions, extend disease control, and delay the time to next treatment (TTNT). However, high-quality prospective data remain limited (15).

Clarifying the relationship between key clinical endpoints such as PFS, OS, and TTNT is therefore crucial for optimizing the management of metastatic NSCLC. Building on our previous findings (16), this study aimed to assess the impact of PDRT on oncological outcomes in a cohort of patients with intra- and extracranial oligoprogressive NSCLC. A deeper understanding of these interactions may help refine patient selection, optimize sequencing with systemic agents, and better define the therapeutic role of PDRT in modern NSCLC care.

## 2 Materials and methods

### 2.1 Study design

From January 2020 to October 2025, we conducted a single-center retrospective analysis of patients with NSCLC treated with PDRT to intracranial and/or extracranial sites of oligoprogression following an initial complete response (CR), partial response (PR), or stable disease (SD) after at least one line of systemic therapy, according to RECIST 1.1 or PERCIST criteria (17, 18).

Oligoprogression was assessed through whole-body CT and/or whole-body FDG PET/CT and brain MRI performed every 3–4 months. Extracranial oligoprogression was defined as ≤5 metastases in no more than 3 organs. Intracranial oligoprogression was defined as any number of lesions with a cumulative tumor volume ≤ 14 cc.

All cases were discussed in a multidisciplinary team (MDT) to define the optimal therapeutic approach.

### 2.2 Intervention

PDRT was delivered with the intent to achieve durable local control (LC) at the progressing site(s) and postpone transition to a new systemic therapy line. Patients treated with purely palliative intent were excluded. Dose prescription was planned to maximize the biological effectiveness using an α/β ratio of 10 (BED10), aiming for a target BED10 ≥100 Gy whenever feasible. Organ-at-risk sparing and compliance with dose–volume constraints were prioritized over target coverage.

Radiotherapy was delivered using volumetric modulated arc therapy with coplanar 6–10 MV flattening-filter-free beams on a LINAC platform. Daily image guidance with cone-beam CT was routinely performed.

### 2.3 Follow-up

Patients underwent scheduled clinical and radiologic follow-up. For intracranial disease, contrast-enhanced MRI was performed every 3–4 months. For extracranial disease, contrast-enhanced CT or FDG PET/CT was performed at 3–4-month intervals. Toxicity was assessed at each visit, and adverse events were documented.

### 2.4 Endpoints

To better capture the clinical benefit of PDRT beyond conventional progression-free survival, the primary endpoint was Event-Free Survival (EFS), defined as a composite endpoint including early oligoprogression (<6 months after PDRT), development of multisite progressive disease (>3 new or progressing lesions), or a change in systemic therapy. Progression within 6 months was defined as any radiologically confirmed progression (including oligoprogression) occurring before the 6-month time point. The occurrence of more than three new or progressing lesions was considered indicative of a more diffuse disease pattern progression, deemed unsuitable for further local therapy, regardless of the timing during follow-up.

The endpoint was considered reached when none of these events had occurred by the time of last follow-up or death.

Secondary endpoints included LC, OS, and treatment-related toxicity, graded according to CTCAE v5.0.

### 2.5 Statistical analysis

Univariate and multivariate Cox proportional hazards models were used to evaluate factors associated with clinical outcomes. Variables with a p-value ≤0.10 in the univariate analysis were included in the multivariate model. Hazard ratios (HRs) and 95% confidence intervals (CI) were reported.

Statistical analyses were performed using MedCalc^®^ v22.021 (MedCalc Software Ltd, Ostend, Belgium). A p-value ≤0.05 was considered statistically significant. Kaplan–Meier (KM) curves were used for descriptive survival analyses. Time-to-event outcomes were calculated from the start date of PDRT.

## 3 Results

A total of 87 patients with a median follow-up of 14 months (range: 1–68) were analyzed. Patient, tumor and treatment characteristics are summarized in **Table 1**. PDRT was delivered across different anatomical sites: 21 treatments (24.1%) targeted the primary tumor, 17 (19.6%) the regional lymph nodes, and 49 (56.3%) distant metastatic lesions, including 15 intracranial lesions (17.2%). At the time of oligoprogression, 35 patients (40.2%) were on chemotherapy, 39 (44.9%) on immunotherapy, and 13 (14.9%) on targeted therapy. The median BED10 for the entire cohort was 60 Gy (range: 28–151.2), with values ranging from 75 Gy (39–151.2 Gy) for primary tumors to 43.2 Gy (28–71.4 Gy) for regional nodes, and 65.1 Gy (28–151.2 Gy) for metastatic lesions. Overall, treatment selection and dose prescription reflected lesion location, proximity to organs at risk, and the underlying clinical intent.

**Table 1:** Patients, tumor and treatment characteristics.

Variable	Data
Age	
Median	68 (range: 43 – 87)
Gender	
Male	50 (57.5%)
Female	37 (42.5%)
Smoking habit	
No	16 (18.4%)
Yes	17 (19.6%)
Former	53 (60.9%)
Unknown	1 (1.1%)
Performance status	
ECOG 0-1	62 (71.2%)
ECOG 2	16 (18.4%)
ECOG 3	9 (10.4%)
Histology	
Adenocarcinoma	75 (86.2%)
Squamous cell	12 (13.8%)
Mutations	
Yes	18 (20.7%)
No	69 (79.3%)
PDL1	
Positive:	50 (57.5%)
< 50%	24 (27.6%)
≥ 50%	26 (29.9%)
Negative	37 (42.5%)
Synchronous	
Yes	43 (49.4%)
No	44 (50.6%)
Systemic therapy	
Chemotherapy Platinum based Taxanes	35 (40.2%) 31 2
Gemcitabine	2
Immunotherapy (Ab antiPD1/PDL1)	39 (44.9%)
Target therapy	13 (14.9%)
Osimertinib	6
Mobocertinib	1
Dabrafenib + Trametinib	2
Alectinib	2
Sotorasib	1
Entrectinib	1
Line of systemic therapy	
1	61 (70.1%)
2	18 (20.7%)
3+	8 (9.2%)
Best response to systemic therapy	
CR	12 (13.8%)
PR	29 (33.3%)
SD	46 (52.9%)
Site of PDRT	
Primary tumor (T)	21 (24.1%)
Regional nodes (N)	17 (19.6%)
Metastatic sites (M)	49 (56.3%)
Number of metastases	
1	70 (80.5%)
2	12 (13.8%)
3	3 (3.4%)
>4	2 (2.3%)
BED10	60 Gy (range: 28 - 151.2)
CTV volume	11.8 cc (range: 0.1 - 386.8)

PDRT, progression-directed radiation therapy; BED10, biologically effective dose with an α/β of 10; CTV, clinical target volume.

### 3.1 Treatment outcomes

#### 3.1.1 Event-free survival

**Figure 1:**
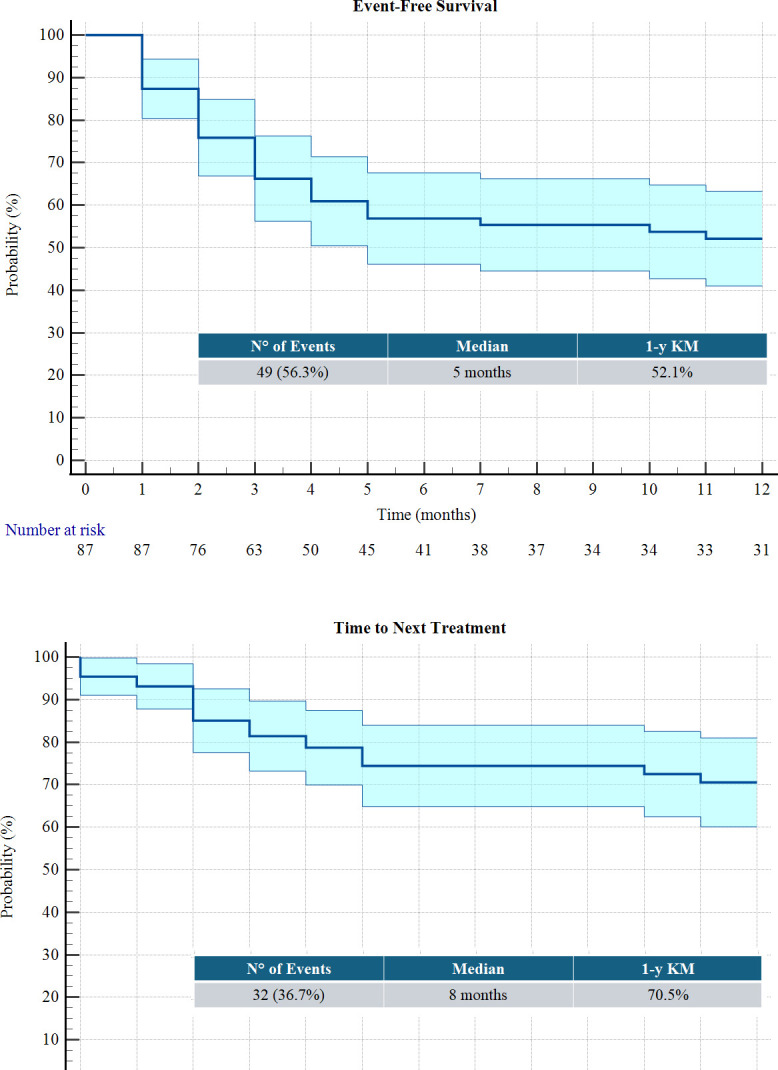
1-year event-free survival and time to next treatment.

A total of 49 patients (56.3%) experienced an oncological event, resulting in a median time to event, death of last follow-up was of 5 months (range: 1–48) and a 1-year KM (**Figure 1**) rate of 52.1% (95% CI: 46.4–57.8). A change in systemic therapy occurred in 32 patients (36.7%), with a median TTNT of 8 months (range: 1–68) and a 1-year KM rate of 70.5% (95% CI: 65.2–75.8). Disease progression was observed in 51 patients (58.6%), although only 17 met the definition of an event according to the study endpoint. Median progression-free survival (PFS) was 5 months (range: 1–39), with a 1-year KM rate of 48.4% (95% CI: 42.5–54.3). Among patients who experienced disease progression, 24 underwent re-irradiation to the site of progression rather than switching systemic therapy.

As shown in **Table 2**, in the univariate Cox analysis, PDRT delivered to the primary tumor was associated with a significantly lower risk of experiencing an oncological event (HR = 0.32, 95% CI: 0.20–0.93; p = 0.04). Patients achieving CR before oligoprogression showed a trend toward improved prognosis (HR = 0.41, 95% CI: 0.14–1.18; p = 0.09), although this did not reach statistical significance. Chemotherapy use was associated with an increased likelihood of experiencing an event (HR = 2.34, 95% CI: 1.23–4.46; p < 0.01) compared to immunotherapy, while larger CTV volumes showed a non-significant trend toward worse EFS. The multivariate analysis confirmed these associations: PDRT to the primary tumor (HR = 0.28, 95% CI: 0.12–0.66; p < 0.01) and CR (HR = 0.31, 95% CI: 0.10–0.95; p = 0.04) were both linked to improved EFS, whereas chemotherapy use (HR = 2.32, 95% CI: 1.20–4.48; p = 0.01) and larger CTV (HR = 1.01, 95% CI: 1.001–1.02; p = 0.03) were significantly associated with worse outcomes.

**Table 2:** Univariate and multivariate Cox regressions for event-free survival.

*Variable*	*Univariate*			*Multivariate*		
	*HR*	*95% CI*	*p-value*	*HR*	*95% CI*	*p-value*
Systemic therapy						
*Immunotherapy* *Chemotherapy* *Target therapy*	Ref. 2.34 1.94	1.22 – 4.45 0.85 – 4.43	***0.009*** 0.11	Ref. 2.32	1.20 – 4.48	***0.01***
Site of PDRT						
*Metastatic sites (M)* *Primary tumor (T)* *Regional nodes (N)*	Ref. 0.43 0.95	0.20 – 0.93 0.45 – 2.01	***0.03***0.90	Ref. 0.27	0.11 – 0.66	***0.003***
CTV Volume	1.01	0.99 – 1.18	*0.08*	1.01	1.001 – 1.02	***0.04***
Response to systemic therapy						
*Stable disease* *Complete response* *Partial response*	Ref. 0.41 1.02	0.14 – 1.18 0.56 – 1.88	*0.09* 0.92	Ref. 0.30	0.10 – 0.94	***0.04***
Age (per 1-year increase)	0.97	0.93 – 1.01	0.13			
Line of systemic therapy						
*1* *2* *≥ 3*	Ref. 1.03 1.88	0.50 – 2.09 0.78 – 4.52	0.89 0.15			
Performance status (ECOG)						
*0-1* *2* *3*	Ref. 1.48 0.91	0.72 – 3.03 0.34 – 2.16	0.27 0.85			
Number of metastases	1.23	0.83 – 1.81	0.28			
Brain disease						
*No* *Yes*	Ref. 1.43	0.68 – 3.00	0.33			
Gender						
*Male* *Female*	Ref. 1.18	0.66 – 2.09	0.56			
BED10	0.99	0.98 – 1.00	0.62			
Histology						
*Adenocarcinoma* *Squamous Cell Carcinoma*	Ref. 1.22	0.54 – 2.73	0.62			
Smoking habit						
*Yes* *No*	Ref. 0.84	0.39 – 1.81	0.66			
Synchronous						
*No* Yes	Ref. 1.11	0.62 – 1.96	0.71			

HR, Hazard Ratio; CI, Confidence Interval; BED_10_, Biologically Effective Dose considering an α/β = 10; V_ctv95%_, Volume of the Clinical Target Volume receiving the 95% of the prescribed dose; V_ptv95%_, Volume of the Plannin Target Volume receiving the 95% of the prescribed dose. Bold are significant variables.

Among patients with intracranial disease (15/87), 9 (60%) experienced an oncological event, with a median time to event of 4 months, with no statistically significant difference compared with patients without intracranial involvement (p = 0.33).

#### 3.1.2 Local control

**Figure 2:**
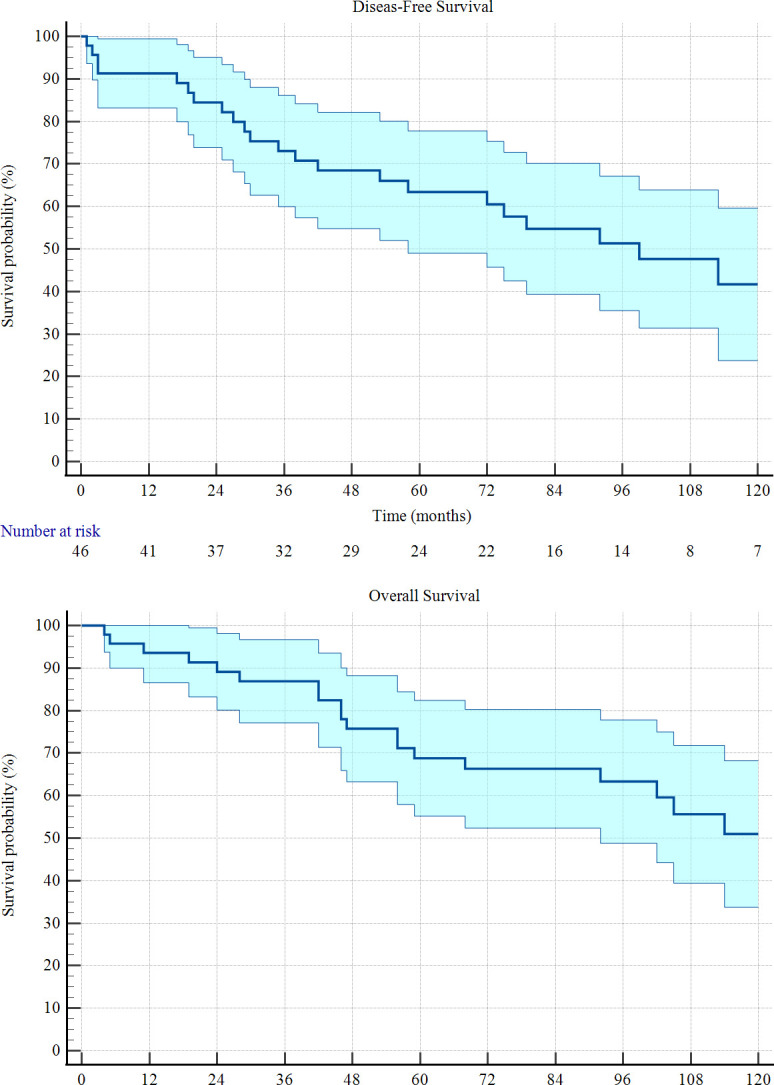
1-year local control and overall survival.

Local recurrence occurred in 18 patients (20.7%), with a resulting median LC of 10 months (range: 1–60). The 1-year LC rate (**Figure 2**) was 81.7% (95% CI: 77.0–86.4). Univariate analysis showed that increasing age was associated with improved LC (HR = 0.95 per year, 95% CI: 0.90–0.99; p = 0.04), and the number of simultaneously treated lesions was independently associated with poorer LC (HR = 1.83, 95% CI: 1.09–3.04; p = 0.02). Both associations were confirmed in the multivariate model, where age remained protective (HR = 0.94, 95% CI: 0.89–0.99; p = 0.02) and the number of simultaneously treated lesions predicted poorer LC (HR = 2.06, 95% CI: 1.20–3.51; p = 0.01).

#### 3.1.3 Overall survival

During follow-up, 34 patients (36.2%) died, with a median OS of 14 months (range: 1–68). The 1-year KM OS rate was 68.1% (95% CI: 62.8–73.4). In the multivariate analysis, several variables were significantly associated with OS. PDRT delivered to the primary tumor correlated with improved survival (HR = 0.20, 95% CI: 0.06–0.73; p = 0.02), while ECOG performance status 2 was strongly associated with higher mortality (HR = 4.62, 95% CI: 1.76–12.12; p < 0.01). Larger CTV volumes (HR = 1.01, 95% CI: 1.01–1.02; p < 0.01) and chemotherapy use (HR = 2.62, 95% CI: 1.04–6.58; p = 0.04) were also predictors of worse OS.

Patients who received re-irradiation rather than a switch in systemic therapy showed a longer median overall survival of 26 months (range, 4–68), with only 7 events (16.7%) recorded.

#### 3.1.4 Safety

Overall, PDRT was well tolerated. Thirteen radiotherapy-related adverse events were observed: eleven cases of radiation pneumonitis, one case of cerebral edema, and one case of brain radionecrosis. All toxicities were mild to moderate (grade 1–2), including nine grade 1 and four grade 2 events, and no grade ≥3 toxicity occurred.

Most toxicities (n = 9) occurred in patients receiving immunotherapy, while two were recorded in patients undergoing platinum-based chemotherapy and two in patients on targeted therapy (dabrafenib–trametinib and alectinib). Specifically, grade 2 pneumonitis developed in two patients on immunotherapy, one receiving chemotherapy, and one treated with dabrafenib–trametinib.

## 4 Discussion

The evolving landscape of systemic oncologic therapies, coupled with advances in imaging, has led to the recognition of oligoprogression as a distinct and meaningful clinical entity. However, evidence on the optimal management of these patients remains limited (19). In our previous series of 40 patients (16), with a median follow-up of 11 months (range: 2–50), PDRT achieved median LC, PFS, and OS of 8 months (range: 1–44), 5 months (range: 0–21), and 10 months (range: 1–48), respectively.

In the present study, we expanded our cohort by including patients who developed intracranial oligoprogression and those treated after our previous publication, reaching a total of 87 patients with a median follow-up of 14 months (range: 1–68). Compared with earlier findings, LC and OS improved to 10 months (range: 1–60) and 14 months (range: 1–68), respectively, while PFS remained stable at a median of 5 months (range: 1–39). These findings suggest that patients who achieve sustained LC tend to maintain broader oncologic stability over time, and that the improvement in LC parallels the extension in follow-up time. Conversely, the persistently limited PFS highlights that many patients still experience early systemic progression at new sites. Importantly, the inclusion of patients treated for brain metastases did not appear to negatively affect clinical outcomes.

To better describe the clinical benefit of PDRT beyond conventional PFS, we also analyzed EFS, a composite endpoint defined as early oligoprogression (<6 months post-PDRT), overt multisite progressive disease, or a change in systemic therapy. This endpoint was designed to more accurately capture PDRT failure compared with TTNT, as it considers both the delay in initiating a new systemic therapy and progression leading to either re-irradiation or treatment discontinuation due to clinical deterioration. In this expanded series, median EFS was 5 months (range: 1–48), while median TTNT was 8 months (range: 1–68), which reinforce the role of PDRT as a meaningful treatment-deferring strategy in patients with oligoprogressive disease.

Franceschini et al. (20) reported that adding RT to standard care (SOC) in oligoprogressive NSCLC resulted in PFS ranging from 5.5 to 10.9 months. Conversely, Mavrikios et al. (11), in a comprehensive review, noted that most available data are retrospective and often limited to specific systemic therapy settings. However, their generalizability remains limited due to the heterogeneity of treatment contexts. The first randomized phase II trial on oligoprogression, by Tsai et al. (21), compared SOC alone versus SOC plus SBRT in patients with oligoprogressive breast cancer or NSCLC. Most SBRT regimens delivered 27–30 Gy in three fractions or 30–50 Gy in five fractions. Among NSCLC patients (28 SOC vs. 31 SBRT), PFS was significantly longer with SBRT (10.0 vs. 2.2 months; HR 0.41, p = 0.0039), although grade ≥2 toxicity occurred in 62% of cases, including three grade 4 events. Similarly, Schellenberg et al. (22) randomized 90 patients with oligoprogressive disease (44% NSCLC) to receive SBRT + SOC or SOC alone. Despite improved LC with SBRT (71% vs. 39%, p = 0.002), no significant differences were observed in DFS (8.4 vs. 4.3 months), OS (31.2 vs. 27.4 months), or TTNT (10.3 vs. 7.6 months). Importantly, severe toxicity (grade >2) remained rare (3.3%). In a retrospective analysis of 168 patients with oligoprogressive or oligorecurrent NSCLC, Ebadi et al. (23) reported median PFS, TTNT, and OS of 6.6, 9, and 31 months, respectively, with grade 3 toxicity in only 1.8% of patients.

Our results are consistent with existing literature, confirming that PDRT yields comparable efficacy to prior studies. Nevertheless, careful patient selection remains crucial for maximizing its benefit. From our cohort, a lower disease burden clearly correlated with better outcomes: larger CTV were associated with poorer EFS and OS. Yamamoto et al. (24) analyzed 1,378 patients with various oligometastatic tumors and demonstrated that each 1-cm increase in CTV diameter worsened OS (HR 1.266; 95% CI: 1.131–1.417; p < 0.001). The LaIT-SABR study (25) also showed that lung oligometastases >20 mm were associated with inferior DFS, highlighting the prognostic relevance of lesion size across disease sites.

In our series, achieving a CR before oligoprogression was another favorable prognostic factor for EFS. Salvador-Coloma et al. (26) found that radiological CR, per RECIST, was significantly associated with prolonged PFS and OS in 360 NSCLC patients treated with targeted therapy. Similarly, a meta-analysis by Rosner et al. (27) demonstrated that CR improved both OS (HR 0.50; 95% CI: 0.45–0.56) and PFS (HR 0.46; 95% CI: 0.37–0.57). These findings collectively support the concept that deep responses before the emergence of oligoprogression may reflect more favorable tumor biology.

The site of progression appeared to influence patient outcomes. Progression confined to the primary tumor correlated with longer EFS and OS compared with progression in lymph nodes or distant sites, indicating that localized relapse may predict a more favorable prognosis even in metastatic settings. This gradient in outcomes according to progression site has been documented across different tumor types: Franzese et al. (28) reported poorer OS for non-lung metastases (HR 1.67; p = 0.02) in 270 colorectal cancer patients, while Franceschini et al. (29) found improved OS for nodal involvement (HR 0.44; p = 0.005) in a cohort of 358 oligometastatic cases. Consistently, Ebadi et al. (23) reported that patients with 3–5 progressive lesions had shorter TTNT and OS compared with those with ≤2 sites of progression, further highlighting the prognostic value of disease burden and anatomical distribution.

Regarding systemic therapy, chemotherapy was associated with higher event rates than immunotherapy. This aligns with the retrospective study by Kim et al. (30), which demonstrated that the introduction of immunotherapy improved OS and TTNT in a large real-world cohort of over 10,000 patients. Conversely, in our series, the line of systemic therapy was not significantly correlated with oncological outcomes, although prior studies have demonstrated such associations (31–33). Kroeze et al. (32) and Mok et al. (33) both identified prior therapy lines as independent predictors of worse PFS in oligoprogressive NSCLC, a finding also observed in the phase II CURB trial (21).

The median BED in our cohort was 60 Gy (range: 28–151.2), which may appear modest compared with ablative regimens (BED10 ≥100 Gy) used in prospective studies (23, 34–36). However, in the oligoprogressive setting, PDRT primarily aims to eradicate resistant tumor clones and prolong the efficacy of systemic therapy rather than achieve radical ablation (37). Thus, higher doses may not always be necessary and could potentially increase toxicity without clear benefit. Our approach favored organ-preserving, stereotactic doses tailored to site and volume, achieving durable control with minimal toxicity. This concept aligns with the findings of Mahmood et al. (38), who reported similar disease control rates between ablative and sub-ablative regimens in 120 patients (59 with NSCLC) progressing under immunotherapy. In support of our approach, we observed low toxicity rates, with adverse events occurring in only 13 patients and never exceeding grade 2, despite the heterogeneity of our population and the variety of systemic treatments administered. Toxicities were more common in patients treated at thoracic sites and in those receiving immunotherapy. This finding is consistent with existing evidence showing that the inflammatory toxicity resulting from the combination of immune checkpoint inhibitors and RT is generally mild, although it may be slightly increased in some settings (39). These immune-mediated interactions may contribute to enhanced early or late inflammatory reactions after RT, particularly in sensitive tissues such as the lung. This underscores the importance of delivering the highest effective radiation dose while strictly respecting organ-at-risk constraints.

At last, it is important to discuss the optimal timing of PDRT, particularly in light of the recently published NORTHSTAR trial (40). This multicenter, single-arm phase II study enrolled 42 patients with advanced EGFR-mutated NSCLC, without restrictions regarding the number, site, or size of metastases. Patients with stable or responding disease after 8 weeks of osimertinib received RT to residual lesions. At a median follow-up of 35.7 months, the study reported a median PFS of 32.3 months, a median OS of 45.0 months, and a median duration of osimertinib of 32.4 months. These findings suggest that integrating RT earlier in the disease course – directed at persistent disease rather than exclusively at sites of progression – may enhance outcomes in selected patients. However, the data remain immature, and results from ongoing randomized trials evaluating similar strategies are eagerly awaited.

### 4.1 Study limitations

The retrospective design inherently introduces selection and reporting biases. The sample size and follow-up duration (median 14 months) are modest, although outcomes at one year remain clinically meaningful in the oligoprogressive setting. Moreover, the inclusion of patients with diverse genomic profiles (wild-type vs. driver mutations) and treated with different therapeutic agents may have influenced the results. Another limitation lies in the novelty of the EFS endpoint, originally introduced in breast cancer (AVATAR Trial (41)), limiting direct comparison with other studies. Nonetheless, some studies have begun to report this endpoint (42), and we consider this a strength, as EFS provides a more comprehensive measure of PDRT efficacy by capturing both disease control and the deferral of systemic therapy.

### 4.2 Conclusions

Our findings indicate that PDRT is an effective, safe, and feasible therapeutic option for patients with oligoprogressive NSCLC. Patients with limited disease burden appear to derive the greatest benefit. These results support the integration of PDRT as a complementary strategy within modern systemic treatment algorithms. Further prospective randomized studies with longer follow-up are warranted to confirm whether PDRT can consistently delay systemic treatment initiation and improve outcomes in this challenging clinical scenario. Such trials will be critical for defining optimal patient selection, dose strategies, and the actual magnitude of benefit.

## Data Availability

The raw data supporting the conclusions of this article will be made available by the authors, without undue reservation.
